# Adipokines, insulin resistance, metabolic syndrome, and breast cancer recurrence: a cohort study

**DOI:** 10.1186/bcr2856

**Published:** 2011-03-30

**Authors:** Sang Woo Oh, Cheol-Young Park, Eun Sook Lee, Yeong Sook Yoon, Eon Sook Lee, Sang Shin Park, Yuil Kim, Nak Jin Sung, Young Ho Yun, Keun Seok Lee, Han Sung Kang, Youngmee Kwon, Jungsil Ro

**Affiliations:** 1 Center for Obesity, Nutrition, and Metabolism, Department of Family Medicine, Dongguk University Ilsan Hospital, Dongguk University College of Medicine, 814 Siksa-Dong, Ilsandong-Gu, Goyang-Si, Gyeonggi-Do, 410-773, Korea; 2 Department of Internal Medicine, Kangbuk Samsung Hospital, Sungkyunkwan University School of Medicine, 108 Pyung-Dong, Chongno-Gu, Seoul, 110-746, Korea; 3 Department of Breast and Endocrine Surgery, College of Medicine, Korea University, 126-1 Anam-Dong 5-Ga, Seongbuk-Gu, Seoul, 136-705, Korea; 4 Department of Family Medicine, Inje University Ilsan Paik Hospital, 2240 Daehwa-Dong, Ilsanseo-Gu, Goyang, Gyeonggi-Do, 411-706, Korea; 5 Department of Veterinary Integrative Biosciences, College of Veterinary Medicine and Biomedical Sciences, Texas A&M University, College Station, TX 77843-4458, USA; 6 Quality of Cancer Care Branch, Research Institute and Hospital, National Cancer Center, 809 MadAu-Dong, Ilsan-Gu, Goyang-Si, Gyeonggi-Do, 411-769, Korea; 7 Center for Breast Cancer, Research Institute and Hospital, National Cancer Center, 809 Madu-Dong, Ilsan-Gu, Goyang-Si, Gyeonggi-Do, 411-769, Korea

## Abstract

**Introduction:**

Several *in vitro *studies have suggested the effects of adipokines and insulin resistance on breast cancer cell proliferation and survival. However, little is known about the clinical significance of these findings.

**Methods:**

We examined associations between breast cancer recurrence and adiponectin, leptin, insulin resistance, and metabolic syndrome (MetS) in a cohort of 747 patients from 2001 to 2004.

**Results:**

Adjusted hazard ratios showed an inverse trend across the quartiles for serum adiponectin concentration in estrogen receptor (ER)/progesterone receptor (PR) -negative patients (*P *for trend = 0.027) but not in ER/PR-positive patients. Compared to the highest quartile for adiponectin level, the lowest quartile showed a hazard ratio of 2.82 (1.03 to 7.68). Homeostasis model assessment for insulin resistance (HOMA-IR) showed a positive trend for recurrence in the ER/PR-negative group (*P *for trend = 0.087) and a negative trend in the ER/PR-positive group (*P *for trend = 0.081). Leptin did not show any associations (*P *for trend >0.05). A linear trend was observed with the number of components of MetS in ER/PR-negative patients (*P *for trend = 0.044). This association disappeared when adjusted for adiponectin and HOMA-IR.

**Conclusions:**

Adiponectin and HOMA-IR have prognostic significance in breast cancer recurrence and interventions related to these factors may protect against recurrence in ER/PR-negative patients. These findings were not observed in the case of ER/PR-positive patients. Further evaluation of these insignificant associations is needed because it might be biased by adjuvant chemotherapy or other confounders.

## Introduction

Recent studies have suggested that breast cancer is associated with insulin resistance [1-5], metabolic syndrome (MetS) [4,6], and adipokine levels [7-15]. However, the clinical significance of these findings remains controversial because only limited human data have been published, and inconsistent results have been obtained from these data [1-4,6-16]. Another weakness of previous studies is that most were conducted under cross-sectional designs that failed to establish temporal relationships for causality; in these studies, risk factors were assessed at the same time or after the study outcomes were measured.

The evaluation of estrogen receptor (ER) and progesterone receptor (PR) expression status in breast cancer is critical because clinical and biological heterogeneity is associated with these hormone receptors [17]. Adjuvant endocrine therapy, such as with tamoxifen or aromatase inhibitors, is recommended in ER/PR-positive cancer and significantly improves disease-free and overall survival [18]. However, this adjuvant therapy is not applicable in ER/PR-negative cancer, which shows a poorer prognosis than ER/PR-positive cancer. Recently, *in vitro *studies showed that adiponectin inhibits growth and enhances apoptosis in an ER-negative breast cancer cell line [19,20], suggesting that potentiating adiponectin action may serve as a valuable adjuvant therapy for ER/PR-negative cancer. However, the clinical significance of adiponectin remains unclear because of a paucity of human data.

The aim of this cohort study was to clarify the relationship between breast cancer recurrence and adipokines, insulin resistance, and MetS. We also investigated whether these associations may be modified by ER/PR status and other factors.

## Materials and methods

### Study participants and follow-up

We studied a cohort of newly diagnosed breast cancer patients who underwent surgery and consented to provide blood samples at the National Cancer Center Hospital, Korea, between April 2001 and December 2004. Among a total of 856 cases considered for the initial recruitment, 747 patients remained eligible after exclusion for the following: (1) distant metastasis at diagnosis (8 cases), (2) ductal carcinoma *in situ *(70 cases), (3) cancer with unreported ER/PR status (29 cases), (4) male gender (1 case), and (5) non-epithelial origin of cancer (1 case of sarcoma).

Breast cancer recurrence included either local recurrence or distant metastasis. Informed consents were obtained from participants. The diagnosis was verified by reviewing hospital records. In addition, women were considered to have recurrent disease when the cause of death was identified as breast cancer. Two patients were classified as censored cases because their deaths were not related to breast cancer. This study protocol was approved by the institutional review board of the National Cancer Center (IRB Protocol No. NCCNCS-09-220).

### Clinical evaluation and definitions

Data collected in baseline evaluations included data regarding demographic characteristics, personal and family medical history, alcohol consumption, smoking history, number of deliveries, oral contraceptive use, hormone replacement therapy, breast feeding, and age at menarche/menopause. Height and weight were directly measured using a standardized protocol. Body Mass Index (BMI) was calculated as follows: weight (kg)/height squared (m^2^). Blood pressure was measured using an automated oscillometric blood pressure device (Colin BP-8800; Colin Corporation, Hayashi, Japan) after the subject had rested for five minutes.

We used the definition of MetS as proposed by the American Heart Association and the National Heart, Lung, and Blood Institute [21]. These criteria require at least three of the following components: abdominal obesity, triglycerides ≥150 mg/dl or receiving drug treatment, HDL cholesterol <50 mg/dl for women or receiving drug treatment, systolic/diastolic blood pressure ≥130/85 mmHg or receiving drug treatment, or fasting glucose ≥100 mg/dl or receiving drug treatment. In this study, we used a BMI ≥25 kg/m^2 ^to define obesity, because waist circumference was not measured at the baseline evaluation.

### Immunohistochemistry

For the assessment of ER and PR expression status, immunohistochemical staining was performed using tissue sections cut from formalin-fixed, paraffin-embedded representative breast tumors. Staining was performed using the I-View DAB detection kit and a Ventana ES autostainer (Ventana Medical Systems, Tucson, AZ, USA) using primary antibodies against ER and PR (both from Ventana Medical Systems). Specimens were defined as ER- or PR-positive when nuclear staining was observed in at least 10% of tumor cells tested [22,23].

### Laboratory assessments

Venous blood samples were taken in the morning following an overnight fast and after a supine rest. After centrifugation, sera were collected and frozen at -70°C until analysis. Blood glucose levels were measured via a hexokinase enzymatic reference method using a TBA-200FR NEO biochemical analyzer (Toshiba, Tokyo, Japan) with a coefficient of variation (CV) of 1.3%. Triglyceride levels were measured via an enzymatic colorimetric method, and HDL cholesterol was measured via a selective inhibition method using Hitachi 7600-210 and Hitachi 7180 biochemical analyzers (Hitachi, Tokyo, Japan) with CV of 3.0% for triglycerides and 5.0% for HDL cholesterol. Plasma insulin levels were measured using an immunoradiometric assay (Biosource, Nivelles, Belgium) with a CV of 1.9%. The homeostasis model assessment for insulin resistance (HOMA-IR) was used to estimate insulin resistance as determined by the following formula: ((fasting insulin (μU/mL) × fasting glucose (mmol/liter))/22.5 [24]. Serum estradiol was measured using an electrochemiluminescence immunoassay analyzer (Roche Modular Analytics E170; Roche, Mannheim, Germany) with a CV of 2.4%. Serum adiponectin levels were measured using an enzyme-linked immunosorbent assay (AdipoGen, Seoul, Korea) with a CV of 3.5%. Serum leptin levels were also assessed via enzyme-linked immunosorbent assay (ALPCO Diagnostics, Salem, NH, USA) with a CV of 4.6%.

### Statistical analyses

The ER/PR status was classified into two categories. Patients having ER-negative and PR-negative was designated as the ER/PR-negative group, and ER-positive or PR-positive as the ER/PR-positive group. Because significant interactions were observed between ER/PR status and the primary measures, and because the use of adjuvant endocrine therapy and prognosis varies according to ER/PR receptor status, we analyzed data according to ER/PR-positive or -negative receptor status.

Patient characteristics according to ER/PR status were summarized as the mean ± standard deviation or percentage and compared using an unpaired *t *test or chi-square statistic, as appropriate. If the distribution of a continuous variable was skewed, median values and interquartile ranges were presented, and the Wilcoxon-Mann-Whitney test was used to detect significant differences.

Owing to skewed distributions and the lack of consensus on cut-off points for discriminating abnormalities, leptin, adiponectin, insulin, HOMA-IR, and estradiol concentrations were categorized as quartiles. These categorizations were defined based on the total sample. For the purpose of illustration, estimates of time to breast cancer recurrence stratified by the quartiles of these variables were displayed using Kaplan-Meier curves. The estimates were analyzed using the log-rank test for trends.

The Cox proportional hazards regression model was used to control for multiple factors simultaneously and to estimate adjusted hazard ratios (HRs) and 95% confidence intervals. The relative increase in the risk of breast cancer recurrence for each of the three higher quartiles was estimated in comparison to that for the lowest quartile; it was also used to test for a linear trend in the HRs across the quartiles. Participants receiving medication for diabetes mellitus were excluded from the insulin and HOMA-IR analyses because these drugs may influence insulin secretion and/or insulin sensitivity. To adjust for possible confounding effects of prognostic factors, we constructed two models. Model 1 was constructed using variables with *P *< 0.25 in the univariate analyses. Model 1 estimated HRs after adjusting for age, alcohol consumption, BMI, regional lymph node metastasis, tumor size, and chemotherapy. BMI was not included as a covariate in the analyses of MetS. To evaluate the independent effects of adipokines and insulin resistance, we constructed model 2 to adjust for the factors in model 1 plus HOMA-IR or adipokine levels, as appropriate.

The proportional hazards assumption was assessed graphically using log-log plots of all independent variables and statistically on the basis of Schoenfeld residuals [25]. No major violations of the proportional hazard assumption were detected. All analyses were performed with Stata version 10.1 (StataCorp, College Station, TX, USA). A two-sided *P-*value < 0.05 was considered statistically significant.

## Results

The median follow-up was 62.2 months (interquartile range, 53.8 to 71.5 months) from the date of the initial breast cancer surgery. In total, 94 patients experienced recurrence: 48 cases (20.6%) were among 233 ER/PR-negative patients and 46 cases (8.9%) were among 514 ER/PR-positive patients. The recurrence rate was 3.69 (cases per 1,000 person-months) for the ER/PR-negative group and 1.44 for the ER/PR-positive group. All patients with ER/PR-positive tumors received adjuvant tamoxifen therapy. Histologically, 681 cases (91.2%) were ductal carcinomas and 66 cases (8.8%) were of other types (lobular, medullary, mucinous, papillary, or tubular carcinoma).

Mean age at inclusion time was 45.9 ± 9.8 (mean ± SD) years and 31.3% were postmenopausal women (Table 1). Most baseline characteristics did not differ between the ER/PR-negative and -positive groups (all *P*s > 0.05; Table 1, some data not shown). However, postmenopausal women were more prevalent in the ER/PR-negative group (*P *= 0.041), and significantly more patients with ER/PR-negative tumors received chemotherapy (*P *< 0.001). Serum adiponectin (*P *= 0.004) and estradiol (*P *< 0.001) levels were higher in ER/PR-positive patients, whereas serum leptin (*P *= 0.039) and HOMA-IR (*P *= 0.013) values were lower. Serum insulin concentrations did not differ between the groups (*P *= 0.179). The frequencies of MetS and its components were distributed evenly between the two groups (all *P*s > 0.05), except for the frequency of elevated triglyceride (*P *= 0.037), which was higher in the ER/PR-negative group.

**Table 1:** Characteristics of study participants, according to estrogen/progesterone receptor status*

Characteristic	All (*N *= 747)	ER/PR-negative† (*N *= 233)	ER/PR-positive† (*N *= 514)	*P*-value‡
**Age (yr, mean ± SD)**	45.9 ± 9.8	46.4 ± 10.6	45.7 ± 9.4	0.336
**Current tobacco use (%)**	3.6	3.4	3.7	0.858
**Alcohol use above a safe level (%)**				
**>0 and <7 g/week**	6.4	7.3	6.0	0.771
**>= 7 g/week**	4.7	4.3	4.9	
**Postmenopausal (%)**	31.3	36.5	29.0	0.041
**Cancer size ≥ 2 cm**	21.6	22.3	21.2	0.732
**Positive lymph nodes**	39.6	38.6	40.1	0.707
**Chemotherapy after surgery**	76.0	87.1	71.0	<0.001
**Adipokines and insulin resistance**				
**Adiponectin, median (μg/ml, IQR)§**	5.9 (4.1 to 8.0)	5.2 (3.6 to 7.5)	6.1 (4.2 to 8.1)	0.004
**1Q**	187	68	119	
**2Q**	187	78	109	
**3Q**	187	35	152	
**4Q**	186	52	134	
**Leptin, median (ng/ml, IQR)§**	6.0 (3.2 to 11.7)	7.8 (3.5 to 12.9)	5.8 (3.2 to 11.2)	0.039
**1Q**	187	53	134	
**2Q**	187	45	142	
**3Q**	187	67	20	
**4Q**	186	68	118	
**HOMA-IR**	1.7 (1.4 to 2.4)	1.9 (1.4 to 2.7)	1.7 (1.4 to 2.3)	0.013
**1Q**	187	52	135	
**2Q**	187	49	138	
**3Q**	186	60	126	
**4Q**	187	72	115	
**Insulin (μU/ml, IQR)§**	6.7 (5.6 to 8.9)	7.0 (5.6 to 9.8)	6.6 (5.6 to 8.6)	0.179
**1Q**	189	63	126	
**2Q**	185	44	141	
**3Q**	186	57	129	
**4Q**	187	69	118	
**Estradiol (pg/ml, IQR)§**	32.3 (14.3 to 89.5)	19.7 (12.7 to 67.2)	39.6 (15.9 to 96.8)	<0.001
**1Q**	190	79	111	
**2Q**	184	65	119	
**3Q**	186	43	143	
**4Q**	187	46	141	
**Components of metabolic syndrome**				
**High blood pressure (%) ∥**	42.0	38.6	43.6	0.390
**Obesity (%)¶**	33.7	36.5	32.5	0.179
Hyperglycemia (%)**	54.4	56.2	53.5	0.616
**Decreased HDL cholesterol (%)††**	60.0	61.8	59.1	0.492
**Elevated triglycerides (%)‡‡**	19.1	23.6	17.1	0.037
**No. of components of metabolic syndrome (%)**				
**1**	25.4	20.2	27.8	0.170
**2**	29.3	30.9	28.6	
**≥3**	35.9	39.1	34.4	

* Plus-minus values are means ±SD.

† Estrogen Receptor (ER) and Progesterone Receptor (PR) status were classified as ER/PR-negative if ER-negative and PR-negative status, and ER/PR-positive if ER-positive or PR-positive status.

‡ *P-*values are for the comparison between ER/PR-negative and ER/PR-positive status. § IQR denotes interquartile range (25th and 75th percentile)

∥High blood pressure was defined if systolic/diastolic blood pressure ≥130/85 mmHg or receiving drug treatment

¶ Obesity was defined if Body Mass Index ≥25 kg/m^2^

** Hyperglycemia was defined if fasting glucose ≥100 mg/dl or receiving drug treatment

†† Decreased HDL cholesterol was defined if HDL cholesterol <50 mg/dl or receiving drug treatment

‡‡ Elevated triglycerides was defined if triglycerides ≥150 mg/dl or receiving drug treatment

**Figure 1:**
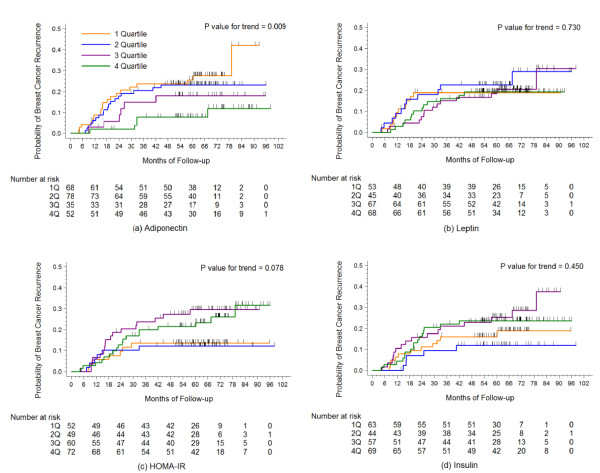
**Kaplan-Meier cumulative recurrence curves for breast cancer patients in the estrogen receptor/progesterone receptor negative group**. The *P*-values for trend were calculated using the log-rank test for trend across the quartiles of adiponectin, leptin, HOMA-IR, and insulin. The hatch marks on the curves indicate times when patients were censored.

**Figure 2:**
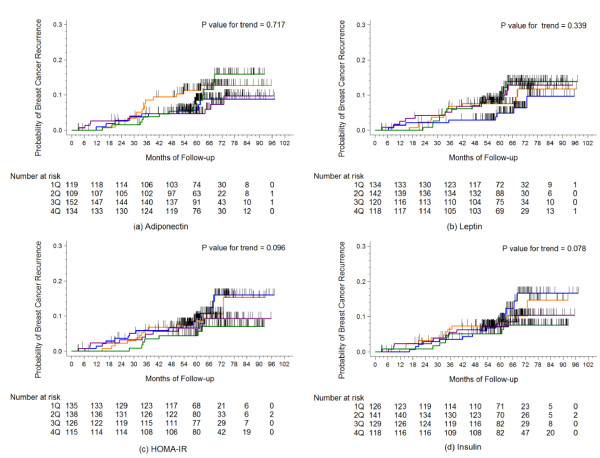
**Kaplan-Meier cumulative recurrence curves for breast cancer patients in the estrogen receptor/progesterone receptor positive group**. The *P*-values for trend were calculated using the log-rank test for trend across the quartiles of adiponectin, leptin, HOMA-IR, and insulin. The hatch marks on the curves indicate times when patients were censored.

In the ER/PR-negative group, serum adiponectin concentrations showed an inverse association with breast cancer recurrence according to the log-rank test for trend (*P *for trend = 0.009; Figure 1). HOMA-IR values showed a positive trend with borderline significance in the ER/PR-negative group (*P-*value for trend = 0.078), whereas insulin levels did not (*P-*value for trend = 0.450). Contrary to this finding, inverse associations for HOMA-IR and insulin with cancer recurrence were observed in the ER/PR-positive group with borderline significance (*P *for trend = 0.096 and 0.078; Figure 2).

**Figure 3:**
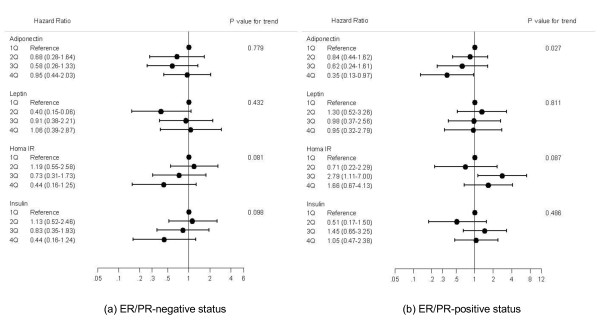
**Adjusted hazard ratio for breast cancer recurrence according to estrogen receptor/progesterone receptor status**. Cox-proportional regression was used for the estimation of hazard ratios with its 95% confidence intervals and *P*-value for trend. Hazard ratios were adjusted for age (<40, 40 to 49, 50 to 59, or ≥60 years), alcohol consumption (none, 0.1 to 6.9, or ≥7.0 g/day), BMI (<23.0, 23.0 to 24.9, or ≥25 kg/m2), regional lymph node metastasis (negative or positive), tumor size (<2 cm or ≥2 cm), and chemotherapy (yes or no).

In the Cox proportional hazards regression analyses adjusting for possible confounders, an inverse linear trend was observed between serum adiponectin concentration and breast cancer recurrence in the ER/PR-negative group (*P *for trend = 0.027 in model 1; Figure 3). This association was further strengthened after adjustment for the effects of insulin resistance (*P *for trend = 0.019 in model 2). Because the graphs are almost identical to each other, the graph for model 2 is not presented in Figure 3. Adiponectin had no impact in the ER/PR-positive group (*P *for trend = 0.779 in model 1 and 0.696 in model 2). HOMA-IR and insulin values showed opposing effects according to ER/PR status. HOMA-IR showed an increasing trend with borderline significance in the ER/PR-negative group (*P *for trend = 0.087 in model 1 and 0.114 in model 2) and a decreasing trend in the ER/PR-positive group (*P *for trend = 0.081 in model 1 and 0.072 in model 2). Likewise, insulin showed an inverse association with borderline significance in the ER/PR-positive group (*P *for trend = 0.098 in model 1 and 0.089 in model 2) but not in the ER/PR-negative group (*P *for trend = 0.486 in model 1 and 0.795 in model 2). Estradiol (data not shown) and leptin did not show any association in either model (*P *for trend > 0.05).

When patients with ER/PR-negative tumors had more components of MetS, the risk of recurrence increased significantly in model 1 (*P *for trend = 0.044; Table 2). However, this association disappeared after adiponectin and HOMA-IR were adjusted in model 2 (*P *for trend = 0.590). In terms of the association between each component of MetS and recurrence, only fasting glucose level in the ER/PR-positive group showed an inverse association with recurrence (*P *= 0.020 in model 1 and 0.058 in model 2).

**Table 2:** Adjusted hazard ratio for metabolic syndrome and its components, according to estrogen/progesterone receptor status

	ER/PR-negative				ER/PR-positive			
	Model 1*		Model 2†		Model 1*		Model 2†	
	Hazard Ratio		Hazard Ratio		Hazard Ratio		Hazard Ratio	
	(95% CI)	*P*-value	(95% CI)	*P*-value	(95% CI)	*P-*value	(95% CI)	*P*-value
**Components of metabolic syndrome**								
**High Blood Pressure‡**	0.86 (0.44 to 1.65)	0.645	0.76 (0.37 to 1.57)	0.457	0.69 (0.36 to 1.31)	0.260	0.88 (0.45 to 1.75)	0.721
**Obesity§**	1.31 (0.72 to 2.37)	0.371	1.01 (0.51 to 2.01)	0.963	0.83 (0.42 to 1.61)	0.573	1.03 (0.50 to 2.12)	0.931
**Hyperglycemia∥**	1.17 (0.65 to 2.12)	0.599	0.81 (0.41 to 1.59)	0.539	0.48 (0.26 to 0.89)	0.020	0.49 (0.24 to 1.03)	0.058
**Decreased HDL cholesterol¶**	1.64 (0.87 to 3.11)	0.128	1.13 (0.56 to 2.28)	0.733	1.06 (0.49 to 2.29)	0.881	1.15 (0.49 to 2.69)	0.750
Elevated Triglycerides**	1.80 (0.93 to 3.47)	0.080	1.17 (0.57 to 2.21)	0.749	1.64 (0.87 to 3.10)	0.129	1.82 (0.89 to 3.72)	0.101
**No. of components of metabolic syndrome**								
**0**	Reference		Reference		Reference		Reference	
**1**	0.81 (0.22 to 2.90)		0.57 (0.15 to 2.15)		2.99 (0.69 to 12.96)		2.28 (0.51 to 10.12)	
**2**	1.09 (0.35 to 3.39)	0.044††	0.67 (0.20 to 2.24)	0.590††	1.68 (0.36 to 7.75)	0.575††	1.85 (0.39 to 8.68)	0.800††
**≥3**	1.95 (0.66 to 5.71)		0.96 (0.29 to 3.18)		1.72 (0.37 to 7.99)		1.95 (0.40 to 9.48)	

*In model 1, hazard ratios were calculated with the use of Cox regression after adjustment for age (<40, 40 to 49, 50 to 59, or ≥60 years), alcohol consumption (none, 0.1 to 6.9, or ≥7.0 g/day), regional lymph node metastasis (negative or positive), tumor size (<2 cm or ≥2 cm), and chemotherapy (yes or no).

†Model 2 was adjusted for the factors in Model 1 plus adiponectin and HOMA-IR.

‡High blood pressure was defined if systolic/diastolic blood pressure ≥130/85 mmHg or receiving drug treatment.

§Obesity was defined if Body Mass Index ≥25 kg/m^2^.

∥Hyperglycemia was defined as fasting glucose ≥100 mg/dl or receiving drug treatment.

¶Decreased HDL cholesterol as defined if HDL cholesterol <50 mg/dl or receiving drug treatment.

**Elevated triglycerides was defined if triglycerides ≥150 mg/dl or receiving drug treatment

††*P*-value for trend was calculated to check the trend of the hazard ratio for breast cancer recurrence according to the number of components of metabolic syndrome.

Because of a possibility of bias from a remnant cancer undetected in the initial evaluation, we tried additional analyses after exclusion of recurrence cases within one year. We found similar significant associations in the additional Cox proportional hazards regression analyses (data not shown). In ER/PR-negative patients, positive trends were observed in adiponectin (*P- *value for trend = 0.035 in model1, 0.020 in model2), HOMA-IR (*P-*value for trend = 0.022 in model 1, 0.035 in model 2), and metabolic syndrome (*P-*value for trend = 0.049 in model 1, 0.691 in model 2). In ER/PR-positive patients, adiponectin and metabolic syndrome did not show any significant trends (all *P *for trend > 0.05) whereas HOMA-IR (*P-*value for trend = 0.022 in model 1, 0.016 in model 2) and insulin (*P-*value for trend = 0.046 in model 1, 0.036 in model 2) showed negative trends.

## Discussion

Based on the relationship between obesity and breast cancer [26], it was hypothesized that adipokines, which are adipocyte-derived peptides, can influence breast cancer occurrence and recurrence. Of the adipokines, adiponectin has drawn keen interest because it is inversely associated with adiposity [27] and is a key regulator of insulin sensitivity and inflammation [28]. In this study, we demonstrated that serum adiponectin levels in ER/PR-negative breast cancer showed an inverse relationship with the risk of recurrence, and the lowest serum quartile level of adiponectin showed a 2.82-fold (1.03 to 7.68) higher risk of recurrence compared to the highest quartile. This association was significant even after adjustment for possible mediating factors, including BMI and HOMA-IR. This finding suggests that assessing adiponectin concentrations may assist in establishing prognosis in ER/PR-negative cancers regardless of obesity and insulin resistance. We also speculate that interventions to increase serum adiponectin levels may represent a therapeutic option for reducing recurrence risk and improving prognosis in ER/PR-negative breast cancer. Candidate interventions may include obesity control; increased physical activity; and pharmacological interventions such as PPAR-γ agonists and others [29].

The mechanism underlying the observed association between adiponectin and breast cancer is not well established. However, insulin-sensitizing, anti-inflammatory, anti-angiogenic, anti-proliferative, pro-apoptotic, and antioxidant mechanisms have been mentioned as possible explanations for the anti-tumor effect of adiponectin [30,31].

In addition, it is unclear why this relationship is only observed in ER/PR-negative cancer. Only four other clinical studies have addressed this issue. One study found a significant association in receptor-negative cancer [12], another in receptor-positive cancer [13], and the remaining two showed no significant association according to receptor status [8,14]. Because of the paucity of reliable evidence, clear explanations for this discrepancy cannot be put forth. The present study focused on cancer recurrence, and all ER/PR-positive patients received adjuvant tamoxifen therapy. Although data regarding the interaction between adiponectin and estrogen are unavailable, there is evidence that estrogen signaling pathways are interrelated with insulin and some adipokines (leptin, tumor necrosis factor-α, and interleukin-6) that have effects on adiponectin production and action [32,33]. From these findings, we postulate that the anti-estrogenic effect of tamoxifen may evoke a differential adiponectin-mediated effect in ER/PR-positive cancer. Therefore, the possible effect of adiponectin in ER/PR-positive breast cancer cannot be excluded based only on the results of this study.

It is interesting to note that contrasting effects for insulin resistance were observed in ER/PR-positive *vs. *ER/PR-negative tumors (*P *for interaction = 0.021). Unexpectedly, an inverse, marginally significant association between insulin resistance and cancer recurrence was observed in the ER/PR-positive group; this trend was also observed in the analyses of serum insulin levels. Furthermore, ER/PR-positive patients with hyperglycemia showed decreased risk of recurrence (Table 2). These findings support the notion of an inverse relationship between insulin resistance and recurrence in ER/PR-positive cancer. The mechanism and clinical significance of these contrary effects are not clear. However, the synergistic effect of estrogen with insulin/IGF-1 [33] and the inverse association between insulin sensitivity and tamoxifen use [34] may at least partly explain these observations. Further study on this issue is required to determine whether the observed effect is due to tamoxifen use, random chance, or a true intrinsic characteristic of ER/PR-positive breast cancer.

Our results regarding MetS are interesting in that the significant association between MetS and recurrence in ER/PR-negative tumors disappeared after adjustment for HOMA-IR and adiponectin in model 2, suggesting that insulin resistance and adipokines mediate the effects of MetS.

This study has several limitations. First, cancer mortality risk was not analyzed because only 55 deaths occurred (7.4% of patients). Second, the effect of human epidermal growth factor receptor 2 status, another important prognostic and predictive factor, was not considered because this parameter was not analyzed in 216 (28.9%) of the patients at recruitment. Third, we used BMI rather than waist circumference to define obesity. Fourth, firm conclusions on the associations of insulin and HOMA-IR with breast cancer recurrences cannot be drawn in the presence of decreased power due to separate analyses by hormonal receptor status. Finally, we did not analyze concentrations of high-molecular-weight (HMW) adiponectin, which is the major source of the active form of this protein [35]. We did not attempt to measure this because the correlation between total adiponectin and HMW adiponectin is high; a recent study found that analyzing HMW adiponectin produced similar results to total adiponectin and did not offer any additional predictive value [10].

Menopausal status and diabetes medications are another issue to be addressed. Because menopausal status and age was highly correlated, we could not include both covariates at the same statistical model simultaneously. But, when we analyzed the model including menopausal status instead of age, we found the same conclusions with the model including age. Nineteen patients were receiving diabetes medication, which could influence blood levels of insulin, glucose, and adiponectin concentration in this study. We tried additional analyses with the exclusion of these patients. And we found similar results with the model including diabetes medication.

This study has several strengths. For example, it highlights temporal-causal relationships. In addition, a number of factors related to obesity and insulin resistance were examined. To our knowledge, this is the first study investigating the complicated effects of obesity, insulin resistance, adipokines, and MetS on the prognosis of breast cancer. Our findings suggest the clinical usefulness of assessing adiponectin, insulin resistance, and metabolic abnormalities in predicting prognosis. The fact that these parameters showed significant associations with ER/PR-negative cancer may assist in the development of new treatment options; improving treatment for these individuals is critical because of the generally poorer prognosis of ER/PR-negative cancer and the inapplicability of adjuvant endocrine therapy.

## Conclusions

This study suggests that measuring serum adiponectin levels and HOMA-IR values has clinical significance in predicting prognosis, and interventions for increasing serum adiponectin level and decreasing insulin resistance may protect against recurrence in ER/PR-negative breast cancer. MetS may also be used to predict prognosis, but this association seems to be mediated by insulin resistance or adipokines. Note that we cannot extend our conclusions to include ER/PR-positive cancer, possibly because of the anti-estrogenic effect of adjuvant endocrine therapy and other possible confounders. Further studies on this issue are needed especially in the ER/PR-positive cancer.

## Abbreviations

BMI: Body Mass Index; CV: coefficient of variation; ER: estrogen receptor; HMW: high-molecular weight; HOMA-IR: Homeostasis model assessment for insulin resistance; HRs: hazard ratios; MetS: metabolic syndrome; PR: progesterone receptor.

## Competing interests

The authors declare that they have no competing interests.

## Authors' contributions

SWO and JR had full access to all of the data in the study and take responsibility for the integrity of the data and the accuracy of the data analysis. SWO, EuSL and YHY were involved in study concept and design. EuSL, KSL, HSK, YK and JR were involved in acquisition of data. SWO, CYP, YSY, EoSL, SSP and JR analyzed and interpreted the data. SWO, YSY and JR drafted the manuscript. SWO, CYP, EuSL, YSY, EoSL, SSP, NJS, YHY, KSL, HSK, YK and JR critically revised the manuscript for important intellectual content. SWO and SSP provided statistical expertise. SWO and YK obtained funding. YK, CYP, NJS and JR provided administrative, technical, or material support. SWO and JR supervised the study.
